# Reconnoitering the Association of Gingival Melanin Pigmentation With Skin Color, Age, and Sex in Pre-School Children of Hazaribag: A Cross-Sectional Study

**DOI:** 10.7759/cureus.30699

**Published:** 2022-10-26

**Authors:** Jaya Verma, Annapurna Ahuja, Vipin Ahuja, Nilima R Thosar

**Affiliations:** 1 Pediatric and Preventive Dentistry, Hazaribag College of Dental Sciences and Hospital, Hazaribag, Jharkhand, IND; 2 Periodontics and Implant Dentistry, Hazaribag College of Dental Sciences and Hospital, Hazaribag, Jharkhand, IND; 3 Pediatric and Preventive Dentistry, Sharard Pawar Dental College and Hospital, Datta Meghe Institute of Medical Sciences (Deemed to be University), Wardha, IND

**Keywords:** pre-school children, melanin, skin tone, dummett-gupta oral pigmentation index, gingival melanin pigmentation

## Abstract

Introduction

The purpose of this study was to compare and evaluate the association of gingival melanin pigmentation with age, sex, and skin tone in pre-school children of Hazaribag, Jharkhand, India.

Materials and methods

The study included 310 children aged 3 to 6 years, who were divided according to skin color into the following: group I: Fair, group II: wheatish, group III: brown; and group IV: dark. The children were selected using a census method where all participants fulfilling inclusion criteria were selected from the outpatient department of the Department of Pediatric and Preventive Dentistry, Hazaribag College of Dental Sciences and Hospital, Hazaribag. The subjects were further grouped into different age groups of 3-4 years, 4-5 years, 5-6 years, and 6 years. Subjects were examined in natural daylight, and gingival melanin pigmentation was assessed using the Dummett-Gupta Pigmentation Index. The scoring was done for each arch segment. These categorical data were analyzed using the chi-square test.

Results

Gingival pigmentation was found to increase with increasing age, whereas female subjects showed less pigmentation than males. Majority of the children had wheatish complexion, and a significant positive correlation was found between skin tone and gingival pigmentation; the darker the skin tone, the darker was the gingival pigmentation. Anatomically, greater pigmentation was found in the anterior than posterior region of arches. Greater pigmentation was found in the labial than lingual region; greater pigmentation was also reported in the mandible than maxilla.

Conclusion

There was a positive correlation of gingival melanin pigmentation with age, sex, and skin tone in pre-school children.

Clinical significance

The association of gingival pigmentation with skin color had been documented, but very few studies have been published on adults and children on this subject. However, there is no study that evaluates the association of gingival melanin pigmentation with age, sex, and skin tone of children of pre-school age.

## Introduction

Human skin color ranges from the darkest brown to the lightest hues. Differences in skin color among individuals are due to variation in pigmentation, which is a result of genetics, exposure to the sun, or both. The actual skin color of different humans is affected by many substances, although the single most important substance is the pigment melanin. Melanin is produced within the skin in cells called melanocytes and is the main determinant of the skin color of darker-skin humans. The gingiva is the fibrous mucosa surrounding the teeth and is the most frequently pigmented part of intra-oral tissues. The deposition of coloring matter, coloration, or discoloration by a pigment pertaining to the gingiva is gingival pigmentation. The knowledge about gingival pigmentation and their etiologies has increased enormously over the past decade. The racial-physiologic pigmentation is not of medical concern but may at times be of esthetic concern. Light brown to black pigmentation may be physiologic or racial in healthy colored-skinned individuals, whereas the same oral pigmentation in Caucasians may be abnormal. The development of pigmentation is regulated by the individual’s genetic makeup. The intensity of pigmentation is frequently altered by physical, chemical, and hormonal factors [1,2].

The ideal color of normal gingiva is coral pink in adults and reddish pink in children. The color of the gingiva is varied among different individuals and is thought to be associated with cutaneous pigmentation. It varies from light to dark brown or black. The skin tone, texture, and color differ in various races and regions. The gingival color depends primarily on the number and size of vasculature, epithelial thickness, degree of keratinization, and pigments within the gingival epithelium. Melanin, carotene, reduced hemoglobin, and oxy-hemoglobin are the prime pigments contributing to the normal color of gingiva, out of which melanin shows the maximum incidence rate [3]. The severity of melanin pigmentation depends on a variety of factors, mostly the megaloblastic activity, and varies from person to person. The Indian population has various color shades ranging from fair to dark. Different patterns of melanin pigmentation can be observed in different Indian populations. Several factors such as amalgam restoration adjacent to gingiva, melanoma, long‑term usage of antimalarial drugs and minocycline, and genetics are potential confounders [4].

The association of pigmentation of gingiva with a positive correlation with skin color in adults has been documented, and the maximum pigmentation was reported to be seen in attached gingiva. Till date, a handful of studies have been published on adults and children related to this subject. However, there is no study that evaluates the association of gingival melanin pigmentation with age, sex, and skin tone of children of pre-school age. Hence, this study was an elusive attempt to relate distribution and intensity of melanin pigmentation in gingiva of young children of Hazaribag town.

## Materials and methods

The present study was carried out in the Department of Pediatric and Preventive Dentistry, Hazaribag College of Dental Sciences and Hospital, Hazaribag, Jharkhand, India. Prior to the start of the study, the protocol for the present study was approved by the Institutional Ethical Committee of Hazaribag College of Dental Sciences and Hospital, affiliated to Vinoba Bhave University, Hazaribag. A total number of 310 children aged 3 to 6 years were selected from the outpatient department of Pediatric and Preventive Dentistry, Hazaribag College of Dental Sciences and Hospital. The children were selected using census method where all participants fulfilling inclusion criteria were selected from the outpatient department of the Department of Pediatric and Preventive Dentistry, Hazaribag College of Dental Sciences and Hospital.

Children aged 3 to 6 years with healthy gingiva and with all primary teeth in the dental arch were included in the study. In addendum to this, consent of the parents/guardians was a mandatory requirement. The exclusion criteria included children with gingivitis, those with periodontitis or with any other systemic diseases, and those with drug or chemical pigmentation on gingiva and with amalgam restorations. Medically compromised children and children with chemical skin peeling and albinism were also excluded.

The children’s demographic details, that is age, gender, and address, were recorded and examined in broad daylight to assess the skin color. The skin color was graded by comparing the color of the inner aspect of the upper arm used as a reference, which is relatively unexposed to sunlight. The skin color of children was recorded under natural light with examiner’s eyes as suggested by Wright [4]. Skin color was recorded as fair, wheatish, brown, and dark by using skin color shade guide. The subjects were divided into four groups according to their skin color in reference to the criteria adapted by Aina et al. as follows [5]:

Group I: subjects with fair skin

Group II: subjects with wheatish skin

Group III: subjects with brown skin

Group IV: subjects with dark skin

This color confirmation was done by inter-examiner calibration, where both the examiners agreed with the specific skin color of the participants. Although there are different techniques to evaluate the skin color, but visual examination of skin color under natural light with eyes is considered as the final judge of color as suggested by Wright [4]. However, in addendum to this, we have used skin color shade guide to grade the skin color in a standardized manner. Intraoral photographs were obtained with a digital camera, and were reproduced with similar sizes to that of actual mouth on a computer display. The distribution and intensity of melanin pigmentation with reference to age, sex, and skin tone were recorded and analyzed by using oral pigmentation index given by Dummett [6].

The Dummett-Gupta Oral Pigmentation Index (DOPI) assessment represents the index that is the assignment of a composite numerical value to the total melanin pigmentation seen on clinical examination of the various tissues comprising the oral cavity. In the case of the gingiva, the assessment is made for each arch separately, and is obtained by dividing the sum of the assigned estimates of pigmentation in the lingual and buccal unit spaces by the total number of unit spaces in the arch. The gingivae of the maxillary and mandibular arches were each divided into 20 unit spaces - 10 on the lingual aspect and 10 on the buccal/labial surfaces - as we took deciduous dentition consisting of 10 teeth in each arch. Each unit space approximates the area of the marginal gingiva and extends from the gingival crest apically to the level of the attached gingiva. The method consists of assigning a numerical oral pigmentation estimate to each one of these 20 unit spaces. The assigned estimate is based on the following scale:

0 = No clinical pigmentation (pink tissue)

1 = Mild clinical pigmentation (mild light brown color)

2 = Moderate clinical pigmentation (moderate brown or mixed pink and brown coloration)

3 = Heavy clinical pigmentation (deep brown or blue-black tissue)

The higher the number, the higher the intensity of pigmentation.

Following the assignment of ratings, the numerical estimates in the maxillary arch were totaled and divided by 20. The resulting number was the DOPI assessment score for the maxillary arch. The mandibular arch was estimated in a similar manner.

DOPI assessment formulae:

(Maxillary gingiva) Sum of assigned estimates of components divided by 20 unit spaces

The DOPI assessment is scaled according to the following designations:

Score 0, no clinical pigmentation; score 0.031-0.97, mild pigmentation, Score 1.00-1.9, medium pigmentation; score 2.00-3.00: heavy pigmentation.

Statistical analysis

Based on the DOPI, the severity of oral pigmentation was categorized and hence children were distributed according to their corresponding severity. These categorical data were analyzed using the chi-square test (test of association). For all tests, a p-value of 0.05 or less was considered for statistical significance.

## Results

A total of 310 children were examined for the study, in which 15.5% were under 3-4 years of age, 20.3% were 4-5 years of age, 24.8% were 5-6 years of age, and 39.4% were under 6 years. Of the total participants, 159 were males and 151 were female, and it was found that all of the children showed gingival melanin pigmentation, thus concluding a 100% incidence of pigmentation. Out of the total subjects, 45.2% were wheatish in color, 23.5% were brown, 19.0% were fair, and 12.3% were dark.

Table 1 illustrates the comparison of gingival melanin pigmentation in percentages among four different age groups and inter-group comparison among mild, medium, and heavy gingival pigmentation. In the age group of 3-4 years, 66.7% of the sample showed mild gingival pigmentation, 33.3% showed medium gingival melanin pigmentation, and none showed heavy gingival melanin pigmentation. In the age group of 4-5 years, 50.8% sample showed mild pigmentation, 41.3% showed medium gingival melanin pigmentation, and only 7.9% showed heavy gingival melanin pigmentation. In the age group of 5-6 years, 53.2% of the sample showed mild gingival pigmentation, 46.8% showed medium gingival melanin pigmentation, and none showed heavy gingival melanin pigmentation. In those aged 6 years, 46.7% of the sample showed mild gingival melanin pigmentation, 53.3% showed medium gingival melanin pigmentation, and none showed heavy gingival melanin pigmentation. When the age groups of 3-4 years, 4-5 years, 5-6 years, and 6 years were compared to each other, it was concluded that medium melanin pigmentation was found to increase with age and that the results obtained were highly statistically significant.

**Table 1:** Total gingival melanin pigmentation in relation with age X^2^ = 25.65, p < 0.001, highly significant

Total		Age (years)				Total
		3-4	4-5	5-6	6	
0.03-0.9	No.	32	32	41	57	162
	%	66.7%	50.8%	53.2%	46.7%	52.3%
1.00-1.9	No.	16	26	36	65	143
	%	33.3%	41.3%	46.8%	53.3%	46.1%
2.00-3.0	No.	0	5	0	0	5
	%	-	7.9%	-	-	1.6%
Total	No.	48	63	77	122	310
	%	100%	100%	100%	100%	100%

Table 2 illustrates the comparison of gingival melanin pigmentation in percentages among gender (male and female) with mild, medium, and heavy gingival pigmentation. In the male group, 48.4% of samples showed mild gingival pigmentation, 48.4% showed medium gingival melanin pigmentation, and only 3.1% showed heavy gingival melanin pigmentation. In the female group, 56.3% of the sample showed mild gingival pigmentation, 43.7% showed medium gingival melanin pigmentation, and none showed heavy gingival melanin pigmentation. When the male and female groups were compared to each other, it was inferred that mild gingival melanin pigmentation was seen more in females than in males, with a statistically significant value. However, medium gingival melanin pigmentation was seen more in males when compared to females.

**Table 2:** Gingival melanin pigmentation in relation to gender X^2^ = 6.04, p = 0.05, significant

Total		Gender		Total
		Male	Female	
0.03-0.9	No.	77	85	162
	%	48.4%	56.3%	52.3%
1.00-1.9	No.	77	66	143
	%	48.4%	43.7%	46.1%
2.00-3.0	No.	5	0	5
	%	3.1%	-	1.6%
Total	No.	159	151	310
	%	100%	100%	100%

Table 3 illustrates the comparison of gingival melanin pigmentation in percentages among four different skin tone groups and inter-group comparison with mild, medium, and heavy gingival pigmentation respectively. In the fair group, 61.0% of sample showed mild gingival melanin pigmentation, 39.0% showed medium gingival melanin pigmentation, and none showed heavy gingival melanin pigmentation. In the wheatish group, 62.9% of sample showed mild gingival pigmentation, 35.0% showed medium gingival melanin pigmentation, and 2.1% showed heavy gingival melanin pigmentation. In the brown group, 38.4% of sample showed mild gingival melanin pigmentation, 58.9% showed medium gingival melanin pigmentation, and 2% showed heavy gingival melanin pigmentation. In the dark group, 26.3% of sample showed mild gingival pigmentation, 73.7% showed medium gingival melanin pigmentation, and none showed heavy gingival melanin pigmentation. When the fair, wheatish, brown, and dark groups were compared to each other, it was found that light-skinned people in the fair and wheatish groups showed more mild gingival melanin pigmentation, whereas when the skin tone becomes darker, intensity of gingival melanin pigmentation becomes darker too and therefore medium-to-heavy pigmentation was seen in brown and dark skinned groups. The results obtained were highly statistically significant.

**Table 3:** Gingival pigmentation in relation to skin tone X^2^ = 27.10, p < 0.001, highly significant DOPI, Dummett-Gupta Oral Pigmentation Index

DOPI		Skin tone				Total
		Brown	Dark	Fair	Wheatish	
Mild pigmentation	No.	28	10	36	88	162
	%	38.4%	26.3%	61.0%	62.9%	52.3%
Medium pigmentation	No.	43	28	23	49	143
	%	58.9%	73.7%	39.0%	35.0%	46.1%
Heavy pigmentation	No.	2	0	0	3	5
	%	2.7%	-	-	2.1%	1.60%
Total	No.	73	38	59	140	310
	%	100%	100%	100%	100%	100%

Table 4 illustrates the comparison of gingival melanin pigmentation of the maxillary anterior region in percentages among four different age groups and inter-group comparison among mild, medium, and heavy gingival pigmentation. When 3-4 years, 4-5 years, and 5-6 years groups were compared to each other, it was concluded that medium melanin pigmentation was found to increase with age and that the results obtained were highly statistically significant.

**Table 4:** Gingival melanin pigmentation of the maxillary anterior region in relation to age X^2^ = 17.64, p = 0.022, significant

Maxillary anterior region		Age (years)				Total
		3 - 4	4-5	5-6	6	
0.03-0.9	No.	22	23	30	45	120
	%	45.8%	36.5%	39.0%	36.9%	38.7%
1.00-1.9	No.	18	26	40	41	125
	%	37.5%	41.3%	51.9%	33.6%	40.3%
2.00-3.0	No.	8	14	7	36	65
	%	16.7%	22.2%	9.1%	29.5%	21.0%
Total	No.	48	63	77	122	310
	%	100%	100%	100%	100%	100%

Table 5 illustrates the comparison of gingival melanin pigmentation of the maxillary posterior region in percentages among four different age groups and inter-group comparison among mild, medium, and heavy gingival pigmentation. When 3-4 years, 4-5 years, and 5-6 years of age groups were compared to each other, it was concluded maxillary posteriors had mostly mild melanin pigmentation and that the results obtained were statistically significant.

**Table 5:** Gingival melanin pigmentation of the maxillary posterior region in relation to age X^2^ = 17.81, p = 0.04, significant

Maxillary posterior region		Age (years)				Total
		3-4	4-5	5-6	6	
0	No.	6	5	6	12	29
	%	12.5%	7.9%	7.8%	9.8%	9.4%
0.03-0.9	No.	37	46	46	76	205
	%	77.1%	73.0%	59.7%	62.3%	66.1%
1.00-1.9	No.	5	9	25	32	71
	%	10.4%	14.3%	32.5%	26.2%	22.9%
2.00-3.0	No.	0	3	0	2	5
	%	-	4.8%	-	1.6%	1.6%
Total	No.	48	63	77	122	310
	%	100%	100%	100%	100%	100%

When Table 4 and Table 5 were compared to each other, it was noted that the gingival melanin pigmentation in the posterior region was lesser than that of the anterior region in the maxilla.

Table 6 illustrates the comparison of gingival melanin pigmentation of the mandibular anterior region in percentages among four different age groups and inter-group comparison among mild, medium, and heavy gingival pigmentation. When 3-4 years, 4-5 years, and 5-6 years of age groups were compared to each other, it was concluded that the mandibular anterior region showed more medium melanin pigmentation and that the results obtained were statistically significant.

**Table 6:** Gingival melanin pigmentation of the mandibular anterior region in relation to age X^2^ = 13.18, p = 0.04, significant

Mandibular anterior region		Age (years)				Total
		3-4	4-5	5-6	6	
0.03-0.9	No.	19	16	18	45	98
	%	39.6%	25.4%	23.4%	36.9%	31.6%
1.00-1.9	No.	24	35	51	53	163
	%	50.0%	55.6%	66.2%	43.4%	52.6%
2.00-3.0	No.	5	12	8	24	49
	%	10.4%	19.0%	10.4%	19.7%	15.8%
Total	No.	48	63	77	122	310
	%	100%	100%	100%	100%	100%

Table 7 illustrates the comparison of gingival melanin pigmentation of the mandibular posterior region in percentages among four different age groups and inter-group comparison among mild, medium, and heavy gingival pigmentation. When 3-4 years, 4-5 years, 5-6 years of age groups were compared to each other, it was concluded that the mandibular posterior region showed milder melanin pigmentation and that the results obtained were statistically significant.

**Table 7:** Gingival melanin pigmentation of the mandibular posterior region to age X^2^ = 44.25, p < 0.001, highly significant

Mandibular posterior region		Age (years)				Total
		3-4	4-5	5-6	6	
0	No.	11	4	6	10	31
	%	22.9%	6.3%	7.8%	8.2%	10.0%
0.03-0.9	No.	27	42	42	83	194
	%	56.2%	66.7%	54.5%	68.0%	62.6%
1.00-1.9	No.	10	7	25	29	71
	%	20.8%	11.1%	32.5%	23.8%	22.9%
2.00-3.0	No.	0	10	4	0	14
	%	-	15.9%	5.2%	-	4.5%
Total	No.	48	63	77	122	310
	%	100%	100%	100%	100%	100%

When Table 6 and Table 7 were compared to each other, it was noted that the gingival melanin pigmentation in the posterior region was less than that in the anterior region in the mandible. Also, when Table 4 and Table 6 were compared to each other, it was noted that the gingival melanin pigmentation in the anterior region of the mandible was lesser than that in the anterior region in the maxilla.

## Discussion

The color of skin and gingiva are two indispensable parameters for an aesthetic smile and a pleasant facial appearance. Today, we know that there are many variations in skin color among members of the same ethnic group and also that changes in the pigmentation of the oral mucosa are not always pathological signs, but may be normal physiologic manifestations. Edwards and Duntley examined skin color spectrophotometrically and found that the color is defined by five primary pigments: melanin, melanoid, oxyhemoglobin, reduced hemoglobin, and carotene [7]. Gingival pigmentation is transmitted genetically in certain populations, and hence it is appropriate to call this pigmentation as physiologic or racial gingival pigmentation. Melanocytes normally occur in the gingiva of all humans [8]. The intensity and distribution of pigmentation of the oral mucosa is variable not only between races but also between different individuals of the same race and within different areas of the same mouth. Physiologic pigmentation is probably genetically determined, but as Dummett and Gupta [9] suggested, the degree of pigmentation is partially related to mechanical, chemical, and physical stimulation, and this variation is related to differences in the activity of melanocytes.

In the present study, the children’s demographic details, that, is age, gender, and address, were recorded and examined in broad daylight to assess the skin color. The skin color was graded by comparing the color of the inner aspect of the upper arm used as a reference, which is relatively unexposed to sunlight. The skin color of children was recorded under natural light with the examiner’s eyes, as suggested by Wright [4]. Skin color was classified as fair skinned, wheatish, brown, and dark using a skin color shade guide. The subjects were divided into four groups according to their skin color in reference to the criteria adapted by Aina et al. as fair, wheatish, brown, and dark. Although there are different techniques to evaluate the skin color, visual examination of skin color under natural light with eyes is considered as the final judge of color as suggested by Wright [4]. However, in addendum to this, we have used skin color shade guide to grade the skin color in a standardized manner. To standardize the calibration, the examiner was capable of seeing the daylight outside the operatory and underwent color vision test to check for color blindness. After checking the vision for color, the skin color assessment was recorded [10]. In the present study, DOPI was used to evaluate the pigmentation of the gingiva [6]. Advantage of using DOPI is that it gives a composite numerical value to total gingival melanin pigmentation. It is used as a clinical tool in estimating the quantitative occurrence of gingival pigmentation and is used as an epidemiologic tool in estimating how widespread gingival pigmentation as well as in comparing the amounts of pigmentation occurring in various oral tissues including gingiva.

Gingival melanin pigmentation and age

In the present study, the children were further grouped into different age groups, 3-6 years, 4-5 years, 5-6 years, and 6 years, and it was found that as the age increased, the intensity of gingival melanin pigmentation also increased, thus showing a positive correlation with age. This observation is in accordance with a study by Kuroda et al. in Japanese children, where it was reported that gingival pigmentation increase up to 6 years of age. Similar findings were observed in the studies by Eaturi et al., which had shown an increase in pigmentation in children of South Indian population from newborns to 15 years of age, and Steigmann, who showed an increase up to 18 years [11-13]. Monash reported increasing pigmentation during the first few years of life in Blacks, which was complete by the end of the second decade of life [14]. Gorsky et al. showed no significant change with age, which is contradictory to the present study and might be due to their exclusion of subjects less than 18 years of age [15]. The increase in the severity of pigmentation from newborn to adulthood (i.e., 0-6 months to 15 years) might be due to physiological growth changes and hormonal changes during pubertal age. The present study observed gingival melanin pigmentation in all the 310 children and is contrary to the report of Prinz who asserted that physiologic pigmentation did not appear in children and was clinically visible only after puberty [16]. Dummett and Gupta reported the presence of pigmentation in newborns as early as 3 hours after birth [9]. The high prevalence reported in the present study when compared to other studies by Dummett, Steigmann, and Amir et al. could be due to the influence of ethnic background on gingival pigmentation. The latter studies included a mixture of fair and dark complexioned population [9,13,17]. In the present study, 310 subjects were involved, and when divided into different age groups, all the age groups showed overall melanin gingival pigmentation.

Gingival pigmentation and racial differences

In the present study, gingival pigmentation of varied intensity was observed in all the participants of the study belonging to Hazaribag city of Jharkhand state, thus inferring a prevalence of 100% in Hazaribag locale. However, this research does not correlate the racial differences as all participants are from the same city. The prevalence of 100% gingival pigmentation in the present study is nearly in accordance with earlier reported studies in Indian races: Joshi and Udhani (100%), Kamat (90%), Pal (90.14%), and Hedin and Axell (96%) [18-21]. Physiologic gingival pigmentation is a characteristic of the darker races, although it is not limited to any one race. Studies by Monash and Dummett in Negroes reported 95% and 92.3% prevalence of gingival pigmentation [9,14]. Van Wyk’s study on South African Bantu tribals reported 98.4% prevalence of gingival pigmentation, Dummett et al, in African-Americans found more than 90% of prevalence, and Hedin and Axell found 91% prevalence of gingival pigmentation in Malays, 70% of Thais, and 74% of Chinese subjects in Malaysia [22-24]. Kuroda et al. reported around 50% prevalence of gingival pigmentation in Japanese school children, and Steigmann in Yeminite Jews found 50% prevalence of gingival pigmentation. Gingival pigmentation was observed in 12.5% of white, 70.4% of yellow, and 93.2% of black Brazilian children [11,13]. Fry and Almeyda also found 5% prevalence of gingival melanin pigmentation in the Caucasoids in Britain. The above studies signify the influence of race (which reflects skin color) and ethnic background on gingival pigmentation [25].

Gingival melanin pigmentation and gender

In the present study, when comparing 161 males and 159 females for gingival melanin pigmentation, the results were statistically significant. An association was found in terms of severity of gingival melanin pigmentation between gender and gingival pigmentation, showing males to have more pigmentation. This association is in accordance with the study by Hedin and Axell who found more pigmentation in males than females in the Chinese population [24]. Contrasting with the present study results, there are findings by Joshi and Udhani, Pal in Indian ethnic population, and other studies by Reade, Bolden, Steigmann, Gorsky et al., Oluwole and Elizabeth, and Eaturi et al., which showed lack of association of gingival melanin pigmentation with gender [12,13,15,18,20,24,26-28]. In the present study, males have reported more gingival melanin pigmentation than females.

Gingival melanin pigmentation and anatomical locations

In the present study, anatomical observation of melanin pigmentation was performed in mandible and maxilla separately, and separate tables for their anterior and posterior tooth region were tabulated. Gingival melanin pigmentation was observed in both labial and lingual aspects of each teeth while determining DOPI assessment score. The present study showed 9.4% of the sample without any pigmentation in the posterior maxillary region and 10.0% of the sample in the posterior mandibular region. In anatomical locations, more number of children in the age group of 3-4 years showed no pigmentation in the posterior region. Thus, the study revealed greater gingival melanin pigmentation in the mandible than maxillary arch, and as we went posteriorly, it was found greater in the anterior than posterior region and more in the labial region when compared to the lingual region. These findings are in accordance with the finding of the study by Eaturi et al., which included individual from newborn to 55 years of age [12]. Raut et al., Pal, Dummett et al., Dummett, Monash, Steigmann, and Hedin and Axell reported greater pigmentation in the anterior than posterior region, labial than lingual [9,12-14,20,21,29-30]. Lesser amount of exposure of the lingual and posterior regions of the mouth due to the effects of light, variations in temperature, the irritating influences of dust particles, and so on could be responsible for the lesser amount of pigmentation in these anatomical locations. In the present study, greater pigmentation was found in the anterior region than the posterior region. Also, greater pigmentation was found in the labial region than the lingual region. Greater pigmentation was also reported in the mandible than maxilla.

Gingival melanin pigmentation and skin tone

In the present study, facial complexion was recorded using skin color shade guide, and the participants were grouped into fair, wheatish, brown, and dark. Out of 310 participants, 140 were of wheatish skin tone that was 45.2%. There was a correlation between the gingival melanin pigmentation and skin tone. In the present study, most of the brown and dark subjects were having medium gingival pigmentation, while most of the fair skinned people showed light gingival melanin pigmentation. As pigmentation of the gingiva is constantly associated with pigmentation of the skin due to similarity between histological structure of the skin and the gingiva, it might be the probable reason for heavy pigmentation in dark complexion people. In general, individuals with fair skin will not demonstrate overt tissue pigmentation, although comparable numbers of melanocytes are present within their gingival epithelium. The melanocytes are generally inactive or hypoactive in such individuals as documented in previous literature [12]. The study done by Dosumu and Dosumu in 2010 showed a statistically significant correlation between facial skin and gingival tissue pigmentation. The subject distribution for facial skin pigmentation was done under two categories, dark and light, as per the criteria developed by Aina et al. in 1978 [31]. Aina et al. developed three categories as dark, medium, and light shades of the skin without any specific clarification and was applied only for Negros population [5]. The present study showed a positive correlation between gingival pigmentation and facial skin complexion, and these findings are in accordance with reports of Eaturi et al., Raut et al., Joshi and Udhani, and Pal, in the Indian population, and other studies by Monash, Dummett et al., Steigmann, Dummett et al., Zimmerman et al., and Oluwole and Elizabeth [9,12-14,18-20,23,28-30,32,33]. On the contrary, studies by Dummett, Kamat, and Patel et al. have not shown any correlation between gingival pigmentation and facial skin complexion.

The results of our study are in contrast with the results of the study conducted by Dummett et al. in 1980, who concluded that there is no correlation between facial skin and the gingival pigmentation [23]. Patel et al. conducted a study on maxillary anteriors to find a correlation between gingival melanin pigmentation and skin tone. This study too showed no association between gingival melanin pigmentation and skin tone [33]. In the present study, a significant positive correlation was found between skin tone and gingival melanin pigmentation. The darker the skin tone, the darker was the gingival pigmentation.

## Conclusions

The present study was an elusive genuine attempt to evaluate and compare the association of age, sex, and skin tone, with gingival melanin pigmentation in pre-school children, and a significant correlation was established after this research was conducted in Hazaribag city of Jharkhand state of East India. The following conclusions are drawn from this study. As the age increased, the intensity of gingival melanin pigmentation also increased, thus showing a positive correlation with age. Males have reported more gingival melanin pigmentation than females. A significant positive correlation was found between skin tone and gingival melanin pigmentation. The darker the skin tone, the darker was the gingival pigmentation. Greater pigmentation was found in the anterior region than the posterior region. Also, greater pigmentation was found in the labial region than the lingual region. Greater pigmentation was also reported in the mandible than maxilla. There is a plethora of literature documented to support these conclusive findings. However, it is difficult to generalize these results over a large population because of various factors such as demographic, ethnic, racial, and genetic differences, yet a definitive hypothesis can be given as the end goal of this study that gingival pigmentation can be positively reconnoitered with age, sex, and skin color. The benefits inferred from this research can be clinically applied to the growing children to perceive gingival pigmentation as a physiological entity and not the pathological one and also it can be depigmented with simple procedures. In addendum to this, further studies are warranted with larger samples over larger areas of different countries to solidify our research outcomes.

The gingival pigmentation in pre-school children of Hazaribag city is significantly associated with age, sex, and skin color. The intensity of gingival melanin pigmentation increases with increasing age, with more male preponderance than females, with the anterior region more affected than the posterior region of the oral cavity. Also, greater pigmentation was noted in the labial region when compared to lingual segment, with more in the mandible than maxilla. The gingival melanin pigmentation was also significantly greater in darker tone children when compared to lighter skin tone children.
